# Companion animal foster caregiving: a scoping review exploring animal and caregiver welfare, barriers to caregiver recruitment and retention, and best practices for foster care programs in animal shelters

**DOI:** 10.7717/peerj.18623

**Published:** 2024-12-13

**Authors:** Grace E. Phillips, Lisa M. Gunter

**Affiliations:** School of Animal Sciences, Virginia Polytechnic Institute and State University (Virginia Tech), Blacksburg, Virginia, United States

**Keywords:** Animal shelter, Companion animal, Animal fostering, Dog, Cat

## Abstract

Each year, millions of animals enter animal shelters across the United States and are met with a variety of potential stressors that can negatively impact their experience, including noise, confinement, and social isolation. Foster care, a unique form of human–animal interaction, is increasingly understood to be an effective tool for improving welfare by allowing animals to escape the stressors of the shelter, providing an environment that allows for greater social interaction, and offering opportunities for improved health and behavior. This review includes 42 published articles, reports, master’s theses, and doctoral dissertations that have previously evaluated companion animal foster care programs. While scientific literature in this area has increased over the last decade, no review of the research exploring companion animal fostering has been published. Here, we examine foster care programs and their effects on human and animal welfare, evaluate the successes and challenges of supporting shelter foster care programs, recommend best practices for programmatic success, illuminate discrepancies in equity and diversity of caregiver engagement, and offer directions for future research in animal foster caregiving. The examinations in this review conclude that fostering provides both proximate (*i.e*., physiological and behavioral) and distal (*i.e*., length of stay and adoption outcomes) welfare benefits for shelter animals as well as their caregivers. Companion animal foster care programs may be further improved by providing greater caregiver support and increasing the diversity and extent of community engagement. Meanwhile, scientific investigations should explore lesser-researched components of foster care programs that are not yet well understood.

## Introduction

Millions of dogs enter animal shelters annually throughout the United States. While many dogs are reunited with their owners or find homes with new adopters, approximately 14% of dogs in U.S.-based shelters are euthanized each year (Woodruff & Smith, 2019; Rowan & Kartal, 2018). Although the number of animals brought to shelters and ultimately euthanized has decreased, lengths of stay for shelter animals are likely increasing as animals await live outcomes, extending their exposure to this stressful environment (ASPCA, 2016; Shelter Animals Count, 2023).

While in the animal shelter, dogs are confronted with numerous stressors, often of greater intensity than those experienced by dogs living in homes, including social isolation, confinement, noise, lack of control, and exposure to new sensory experiences (Dalla Villa et al., 2013; Gunter et al., 2019; Riggio, 2018; Taylor & Mills, 2007). As a result, dogs living in shelters have higher cortisol and lower levels of rest than dogs living in homes (Gunter et al., 2019; Owczarczak-Garstecka & Burman, 2016; van der Laan et al., 2021; van der Laan, Vinke & Arndt, 2023). When living in a home, dogs can move more freely and consistently interact with humans, conspecifics, and other animals. These experiences are often limited for dogs in shelters for their own health and safety (Hubrecht, 1995). Due to these restrictions, chronic social isolation in the shelter is thought to be one of the most profoundly negative experiences for dogs, contributing to their compromised welfare (Beerda et al., 1999a, 1999b; Hennessy et al., 1997).

Volunteers can reduce the social isolation that shelter dogs face by providing them with human as well as conspecific interaction, increasing their enrichment opportunities, and improving their welfare (Gunter & Feuerbacher, 2022). An interaction out of the kennel with a person reduces cortisol levels for dogs living in shelters (Hennessy et al., 1997, 1998, 2002; Coppola, Grandin & Enns, 2006; Hennessy, Morris & Linden, 2006; Bergamasco et al., 2010; Menor-Campos, Molleda-Carbonell & López-Rodríguez, 2011; Shiverdecker, Schiml & Hennessy, 2013; Normando et al., 2006; Dudley, Schiml & Hennessy, 2015; Willen et al., 2017; McGowan et al., 2018). A unique form of this type of human–animal interaction, foster care, provides shelter animals an escape from the many potential stressors in the animal shelter and the opportunity to rest while in the foster home (Fehringer & Dreschel, 2014; Gunter et al., 2019; Hennessy, Morris & Linden, 2006).

As such, foster care programs are increasingly understood to be effective in improving the welfare of shelter-living dogs (Gunter et al., 2019, 2022, 2023). Emerging evidence suggests that fostering companion animals promotes improved emotional, social, and physical well-being for human foster caregivers as well as their fostered companion animals (Powell et al., 2024; Roseveare et al., 2023). While the scientific literature surrounding feline foster care is limited, preliminary evidence suggests that brief fostering stays may not significantly decrease adult feline stress, but foster care may positively affect the health, growth, sociability, and fearfulness of kittens (Berliner et al., 2022; Campbell et al., 2024; Graham, Pearl & Niel, 2024; Vitale et al., 2022). Fostering may also reduce the likelihood of euthanasia for cats and kittens (Berliner et al., 2022; Kerr et al., 2018).

The present review evaluates previous efforts to understand companion animal fostering. While there has been sustained recent research activity on this topic, a review considering both human and animal perspectives has not yet been published. The present review fills this gap, examining 42 published articles, reports, and graduate student theses and dissertations that have investigated companion animal fostering. In addition to examining its effects on human and animal welfare, the scope of this review includes an evaluation of successes and difficulties in sustaining foster care programs, recommendations of best practices for foster care programs, discrepancies in equity and diversity of foster care engagement, and directions for future research about companion animal foster caregiving. The explorations and recommendations included in this review are intended to aid animal shelters and welfare organizations in the development, growth, and maintenance of their foster caregiving programs. Furthermore, this publication is intended to promote future scientific investigations into the impacts of foster care programs on human and animal welfare.

## Search methodology

The content of this review was produced by reading and summarizing English-language publications, published between the dates of 1990 and July 2024, on the impacts of foster care programs for shelter animals and their caregivers. The sources used in this research were obtained using the following search engines: Google, Google Scholar, and the Virginia Tech University Library Database. Keywords and combinations used during article searches included: dog, cat, companion animal, animal fostering, foster care, foster care program, and shelter animal. Review content included peer-reviewed journal articles, chapters in edited books, master’s theses, and doctoral dissertations. A few non-peer-reviewed sources were also included when they provided clarifying data on foster care statistics that could not be found in a peer-reviewed article. These sources were also published during or after 2020 to ensure relevance to the current state of companion animal fostering.

To produce this review, each combination of keywords was initially searched in all three search engines. The most recent search using these keyword combinations was July 8th, 2024. The titles of produced results were read; if the title revealed that the subject of the article was related to animal fostering, animal foster care programs, or shelter animal welfare, the abstract of those articles was also read. If the abstract did not discuss findings related to companion animal foster caregiving (*i.e*., fostering dogs, cats, and or small mammals), the relationship between caregivers and fostered animals, the experience of fostering for caregivers, or limitations to foster caregiving for animal welfare organizations, then that article was not included in the review. Keywords were searched in each combination and search engine until the results were no longer relevant or exceeded 100 sources, depending on which occurred first. The reference section of each relevant source was searched for additional peer-reviewed publications on foster caregiving.

A total of 81 articles, books, dissertations, and non-peer-reviewed sources were included in the background and review of this paper. However, only 42 of these articles were fully reviewed. Titles of the 42 relevant articles were compiled, their contents summarized, and each article was categorized into one or more of the 11 major sections of foster caregiving that constitute the body of this review based on their subject matter: (1) proximate effects of fostering on companion animal welfare, (2) distal effects of fostering on companion animal welfare, (3) effects of fostering on the physical health of caregivers, (4) effects of fostering on the mental wellbeing of caregivers, (5) attachment and foster caregiving, (6) developing foster care programs in animal shelters, (7) evaluating foster care programs, (8) changes to foster programs during the COVID-19 pandemic, (9) challenges and obstacles in foster caregiving, (10) discrepancies in equity and diversity of foster care programs, and (11) best practices and recommendations for shelter foster care programs (Table 1). We recognize that not all relevant publications may have been included with a bias toward English-language publications.

**Table 1:** Sources included in each section of this review and the subject matter of those sections.

Section number	Title	Sources included in section
1	Proximate effects of fostering on companion animal welfare	Gunter et al. (2019, 2021), Vitale et al. (2022), McCobb et al. (2005), Stella, Croney & Buffington (2014), L Gunter, 2024, unpublished data
2	Distal effects of fostering on companion animal welfare	Mohan-Gibbons et al. (2014), Patronek & Crowe (2018), Gunter et al. (2022, 2023), Berliner et al. (2022), O’Hanley, Pearl & Niel (2021), Graham et al. (2024), Graham, Pearl & Niel (2024), Campbell et al. (2024), Kerr et al. (2018)
3	Effects of fostering on the health of caregivers	Ortmeyer & Robey (2019), Potter et al. (2019), Reese et al. (2024)
4	Effects of fostering on the mental wellbeing of caregivers	Sanderson et al. (2023), Powell et al. (2024), Daily (2021)
5	Attachment and foster caregiving	Topál et al. (1998), Gácsi et al. (2001), Thielke & Udell (2020, 2019), Roemer (2004), Reese (2021), Reese et al. (2022b), DeWitt (2020), Gunter et al. (2023, 2022), Powell et al. (2024), Reese et al. (2024)
6	Developing foster care programs	Ackerman et al. (2023), Daily (2021)
7	Evaluating foster care programs	Gunter et al. (2023), Reese et al. (2024, 2022b), Patronek & Crowe (2018)
8	Changes to foster programs during the COVID-19 pandemic	Gunter et al. (2022), Reese et al. (2022a), Morgan et al. (2020), Powell et al. (2021), Carroll, Reeve & Torjussen (2024)
9	Challenges and obstacles in foster caregiving	Gunter et al. (2019, 2022), Roemer (2004), Reese et al. (2022b, 2024), DeWitt (2020), Daily (2021), Reese et al. (2024), Chur-Hansen et al. (2014), Taraciuk et al. (2020), Powell et al. (2024), Gunter et al. (2023)
10	Discrepancies in equity and diversity of foster care programs	Gunter et al. (2022), Graham et al. (2024), Roemer (2004), Reese et al. (2022b), Daily (2021), Taraciuk et al. (2020), McLennan et al. (2022), Toohey & Krahn (2018), Ly, Gordon & Protopopova (2021), Gandenberger et al. (2021), McDonald et al. (2022), Neumann (2010)
11	Best practices and recommendations for foster care programs	Gunter et al. (2019, 2023), Ortmeyer & Robey (2019), Powell et al. (2024), Potter et al. (2019), Sanderson et al. (2023), Thielke & Udell (2020), Roemer (2004), Reese et al. (2022b)

### Proximate effects of fostering on companion animal welfare

Foster caregiving provides animals with housing, husbandry, social interaction, and other forms of enrichment. Some of the first foster care programs in the United States were initiated in the 1980s as animal shelters attempted to improve the care they provided and save the lives of more companion animals that were homeless (Houser, 2018). Following the end of the 20th century, foster care programs were more commonplace in shelters across the United States compared to years prior. As foster programs increased in popularity across the United States, Gunter et al. (2019) examined the effects of one and two nights of foster care on indicators of welfare with 207 dogs living in one of five U.S. shelters. These indicators included dogs’ cortisol levels, a hormone implicated in the stress response system, as well as their bouts of uninterrupted rest, as dogs in shelters had previously been shown to spend less time at rest than dogs in homes (Owczarczak-Garstecka & Burman, 2016).

Gunter et al. (2019) found that the dogs’ cortisol: creatinine ratios significantly declined during their short-term fostering stays but increased to pre-fostering levels upon returning to the animal shelter. Dogs also had longer periods of uninterrupted rest in foster homes as compared to their time in the shelter before fostering; in some cases, dogs’ rest demonstrated a carryover effect of the intervention with continued longer bouts of rest in the shelter following foster care. These findings presented promising evidence for temporary fostering as a means to positively affect the immediate welfare of shelter dogs awaiting adoption.

As time spent outside of the animal shelter emerged as an evidence-based intervention for improving shelter dog welfare, Gunter et al. (2021) measured changes in cortisol and activity levels of 164 shelter dogs before, during, and after a 2.5-hour outing with a caregiver from one of four U.S. shelters. Brief outings, if beneficial in reducing stress and increasing rest comparable to temporary fostering, would be a more likely intervention to be carried out by animal shelters and their caregivers given their shorter duration. During these outings, the authors found that approximately three-quarters of dogs and their caregivers walked, hiked, jogged, or engaged in some sort of outdoor activity.

In the analysis of their physiological data, Gunter et al. (2021) found that dogs’ stress levels were higher during their outings; dogs’ cortisol and activity levels returning to baseline (pre-outing levels) the following day. Moreover, minutes that the shelter dogs spent engaging in high-intensity activity increased while minutes spent resting decreased during their outings. Although cortisol: creatinine ratios were elevated during these outings, increases in cortisol alone are not necessarily indicative of reduced welfare. Instead, they may be associated with greater arousal as indicated by engagement in more physically intense activity.

Nevertheless, the analysis of dogs’ elevated cortisol levels conducted by Gunter et al. (2021) accounted for the dogs’ activity throughout the study, leading to their conclusion that increases in cortisol during the outing were likely due to the physical as well as the psychological stress of the outing, such as traveling to novel locations with their caregiver and perceiving a variety of unfamiliar people and dogs. During temporary fostering, dogs likely experience psychological stress as well when first entering their foster home as they habituate to the new environment. However, the longer period of caregiving, in which the dogs remain overnight and are able to rest, may be related to stress-relief that Gunter et al. (2019) observed that was not found by Gunter et al. (2021). Less than one-quarter of caregivers that provided a brief outing spent time exclusively in a home with their shelter dog.

Weeklong fostering of dogs from animal shelters has been shown to affect dogs in ways similar to temporary fostering of one and two nights. In a study by L Gunter (2024, unpublished data), the authors found the cortisol: creatinine ratios and activity levels of 84 dogs at two shelters in the U.S. (a municipal shelter and a nonprofit with municipal contracts) were positively influenced by seven days of foster care. Dogs’ cortisol levels were lowest when living in a home as compared to their time in the shelter with no significant difference in cortisol before and after fostering. Additionally, Gunter et al. observed that dogs spent the most time in the lowest activity levels, including rest, when they were living with foster caregivers. In total, these findings suggest that longer durations of foster care in a home are beneficial for dogs without detrimental effects on their welfare upon return to the animal shelter.

While most research about foster programs has focused on shelter dog welfare, a study by Vitale et al. (2022) expanded our understanding of such interventions with shelter cats. In contrast to findings about fostered dogs by Gunter et al. (2019; L Gunter, 2024, unpublished data), the authors found that both overnight and weeklong fostering stays did not significantly improve cats’ social behavior or stress levels as measured by their urinary cortisol (*n* = 26). Vitale et al. (2022) found that, whether fostered for one week or one night, fostered cats did not score higher on a test of social behavior than cats in the shelter. Specifically, fostered cats did not exhibit higher levels of sociability towards human play and petting when in foster care as compared to the shelter. Additionally, cats’ fear and aggression-related behaviors were not significantly different between the two environments.

While Vitale et al. (2022) did not find an effect of their fostering intervention, their data appear to indicate that more cats had increases in cortisol during overnight fostering than during weeklong fostering, demonstrating more of a mixed effect, although sample sizes were limited. When considering cats’ sensitivity to new environments (as demonstrated in introductory protocols that are often recommended by shelters when bringing home a newly adopted cat), it is possible that longer durations of foster care (*i.e*., ≥ one week) may be needed to improve shelter cats’ proximate welfare (McCobb et al., 2005; Stella, Croney & Buffington, 2014; Vitale et al., 2022).

### Distal effects of fostering on animal welfare

Foster care programs are effective at increasing live outcomes (*e.g*., adoption or transfer to another shelter) for shelter animals (Barnette, 2009; Maubach, 2014; Rand et al., 2018; Reese, 2021), but foster care is often associated with longer stays in the organization’s care than residing solely in the shelter awaiting adoption. This may be related to decreased exposure to individuals interested in adopting while animals reside in foster homes as compared to living in an animal sheltering facility where potential adopters, staff, and volunteers can view and visit.

In their study of foster caregiving at a U.S. Southwest animal shelter, Patronek & Crowe (2018) found that fostered dogs were five times more likely to experience a live outcome, increasing to 20 times for adult dogs. When Gunter et al. (2023) examined the distal effects of brief outing and temporary fostering programs at 51 U.S. animal shelters, they uncovered that dogs that experienced a brief outing with a caregiver, for about three hours on average, were five times more likely to be adopted as compared to dogs that did not experience an outing during their shelter stay. Moreover, shelter dogs that were temporarily fostered, for at least one-and-a-half days, were over 14 times more likely to be adopted. When compared to shelter dogs that did not participate in these interventions, brief outing and temporarily fostered dogs did have significantly longer lengths of stay, but often this greater time in care was prior to their outing or fostering. Following the intervention, brief outing and temporarily fostered dogs’ lengths of stay were comparable to their non-intervention counterparts (10 days).

In their study, Patronek & Crowe (2018) also found that, while less than 90% of shelter dogs had a live outcome (*e.g*., adoption or transfer to another shelter for placement), almost 98% of dogs that experienced foster care left the shelter’s care alive. Upon return from their fostering stay, shelter staff reported a 70% reduction in dogs’ health issues. During the COVID-19 pandemic (see also Section “Changes to Foster Programs During the COVID-19 Pandemic”), Gunter et al. (2022) identified that over 93% of fostered dogs experienced a live outcome, with about 83% being adopted and slightly less than 10% being transferred to another welfare organization for placement. During the four-month data collection period, less than 2% of fostered dogs were euthanized, died at the shelter, or were lost in the shelter’s care (with over 5% remaining in the care of the shelter, often in foster care). Additionally, the authors uncovered that as the age of dogs increased in their study, so too did their lengths of stay in foster care.

Foster-to-adopt programs are a form of foster care in which individuals provide caregiving to animals they are interested in adopting. Unsurprisingly, this form of fostering has the highest likelihood of caregiver adoption. Gunter et al. (2022) found that prospective adopters in these situations adopt their dogs in nearly three-quarters of experiences. Nevertheless, adoption by a caregiver does not occur as often with other types of foster care. Gunter et al. (2023) found that 4% of caregivers who provide brief outings and 12% who temporarily foster adopt their dogs. Meanwhile, 18% of caregivers who fostered for longer durations during the pandemic adopted their dogs (Gunter et al., 2022). Moreover, Gunter et al. (2022) found that new foster caregivers are over four times more likely to adopt their fostered dogs than caregivers with a prior relationship with the shelter. Furthermore, dogs that were adopted by these new foster caregivers often (77%) have no resident dog in their home.

Cat fostering has not received as much attention in the scientific literature as dog foster programs. Berliner et al. (2022) examined the impacts of foster programs on the health and growth rate of kittens, compared to kittens that were raised in catteries, laboratories, and breeding facilities. In their study, shelter kittens under nine weeks of age were enrolled (*n* = 203) and fostered with caregivers trained by shelter staff. Berliner et al. (2022) found that kittens that participated in these programs experienced more live outcomes and increased growth rates than kittens raised in other settings. While preliminary, these results provide promising evidence of the health benefits of kitten foster care programs.

In addition to the physical health effects, researchers have also explored the influence of foster care on the behavior of developing kittens. O’Hanley, Pearl & Niel (2021) investigated the behavioral characteristics of 262 adult cats that were raised in foster care and then adopted. They found that foster care did not impact the likelihood of kittens developing human-, conspecific-, or dog-directed aggression later in life; instead, these behaviors were found to be more associated with specific cat characteristics, the adoptive home environment, and adopter’s training techniques. However, two studies by Graham et al. (2024) and Graham, Pearl & Niel (2024) suggested that, with specialized positive reinforcement training for foster caregivers, limited non-social enrichment, and regular, controlled social stimuli, foster care can lead to increases in human-directed sociability and decreases in kitten fearfulness. Similarly, Campbell et al. (2024) found that a fostering intervention of alone time, social interaction with humans, and access to quiet, social areas was associated with less fearful, more social, and less aggressive kittens.

While most cat-related fostering research has focused on kittens, Kerr et al. (2018) explored the influence of foster caregiving on adult cat outcomes at an animal shelter in Queensland, Australia. Between 2011 and 2016, the number of cats placed into the shelter’s foster care program nearly doubled. During this time, euthanasia for adult cats decreased by 43%, and behavior-related euthanasia by 85%. Moreover, cat adoptions doubled between 2011 and 2016 when compared to the shelter’s previous five years. While these findings from Kerr et al. (2018) are correlational in nature and likely other programmatic changes occurred at the shelter during these years, taken together with the results of the previous studies, foster care programs likely benefit the distal welfare of both kittens and adult cats.

### Effects of foster care on the health of caregivers

The scientific literature has explored the effects of foster care programs on human caregivers. Studies have shown that fostering animals can improve the physiological health, of not only the fostered animal, but also the caregiver. Ortmeyer & Robey (2019) studied changes in health indicators of four veterans providing canine caregiving for a shelter foster program. Before and after fostering a dog, veterans answered questions about their perceived mood, stress, depressive symptoms, and overall health. Additionally, participants wore health monitors for the two-month period of foster care to measure their steps, heart rate variability, and activity levels. Ortmeyer & Robey (2019) found that the veteran foster caregivers took more steps daily and had greater heart rate variability as well as higher levels of positive affect and decreased depressive symptoms after providing foster care. While the results of this study offer promising indications of improved social, emotional, and physical quality of life, it is worth noting that the study did not include a control group of non-fostering veterans. Due to the requisite interactions with researchers and the animal welfare organizations within the study, veterans received increased contact with people, which may have contributed to a higher quality of life, regardless of their canine fostering experiences. Moreover, the study was not longitudinal in design, so it is unknown if the health effects observed during veterans’ initial fostering period (60 days) would have persisted following subsequent caregiving experiences.

Similarly, Potter et al. (2019) used accelerometers to measure the activity levels of 11 dog foster caregivers across 6 weeks. The results of their study indicated that foster caregivers spent significantly less time sitting and more time engaging in higher activity levels during caregiving than they did prior to fostering a dog. In a survey of over 600 U.S. dog foster caregivers, Reese et al. (2024) found that caregivers in their study reported feeling happier and believing that their dog helped them “stay healthy”.

### Effects of foster care on the emotional wellbeing of caregivers

Fostering companion animals can not only enhance the physical health of caregivers, but aspects of social and emotional wellbeing as well. In their study of foster caregiving with older adults (≥60 years of age) living alone in the U.S., Sanderson et al. (2023) found that foster caregivers reported significant decreases in feelings of loneliness when fostering a cat. Another prospective study by Powell et al. (2024) surveyed 131 foster caregivers across five U.S. shelters before, during, and after fostering a shelter animal. Responding caregivers reported that fostering an animal gave them greater companionship, emotional support, and social and physical quality of life. Caregivers’ attitudes were generally positive toward fostering, with 86% reporting that they would foster an animal again. Nevertheless, foster caregiving did not appear to offer significant benefits to caregivers’ mental health (measured through the Positive and Negative Affect Schedule); thus, while foster caregiving likely offers benefits to emotional and social quality of life, it may have less efficacy as a mental health intervention.

Daily (2021) explored the experiences of companion animal foster caregivers, including aspects of fostering they found to be the most emotionally rewarding and fulfilling. In their survey, completed by 85 foster caregivers, many reported that the most rewarding component of caregiving was connecting their dogs with loving families and homes. Daily found that the second most reported emotional reward for caregivers was helping dogs medically recover and grow behaviorally in foster care. Caregivers also indicated experiencing emotional benefits from meeting and working with new dogs and making a difference in the dogs’ lives. Despite over a quarter of surveyed caregivers in the study no longer providing foster care, Daily’s findings suggest that caregiving, in addition to the previously described physical health benefits, can provide individuals with companionship and emotional fulfillment; however, changes in housing and family circumstances can reduce their involvement in foster caregiving (see “Challenges and Obstacles in Foster Caregiving”).

### Attachment and foster caregiving

Previous research has determined that dogs form social attachments with caregivers and owners, much like an infant would with its parent (Parthasarathy & Crowell-Davis, 2006; Prato-Previde et al., 2003; Topál et al., 1998; Gácsi et al., 2001). Three primary infant attachment styles uncovered using Ainsworth’s Strange Situation Test can apply to relationships between dogs and caregivers. These styles include *secure attachment*: the dog is moderately anxious or stressed when separated from the caregiver but is calmed when the caregiver returns, *insecure-avoidant attachment*: the dog does not appear to be stressed or upset when separated from the caregiver and is distant when the caregiver returns, and *insecure-resistant attachment*: the dog is highly upset when separated from the caregiver, and the caregiver’s return does not lower the dog’s distress (Ainsworth & Bell, 1970; Thielke & Udell, 2019).

Research exploring attachment styles has often focused on the relationship between pets and their owners. In these studies that use a modified Strange Situation Test, dogs exhibit a variety of behaviors that indicate a secure attachment style: seeking proximity and physical touch with their owner, willingness to explore an unfamiliar area, searching for their separated owner, and behaving anxiously when separated from their owner. During this latter component of the test, dogs have been found to follow their owner when leaving and then, wait by and scratch or bark at the door. Upon the owner’s return, dogs greet their owners for longer periods of time and more excitedly (*e.g*., jumping, touching clothing, and loose body language) than they would with a stranger (Topál et al., 1998; Gácsi et al., 2001).

A few studies have delved into the types of attachment dogs exhibit during foster caregiving. Thielke & Udell (2020) explored the relationships that fostered dogs had with their caregivers in comparison to owned dog relationships and the attachment styles of non-fostered shelter dogs. They found that fostered dogs showed equivalent proportions of secure attachment to their foster caregivers as dogs do with their owners. Fostered dogs were more likely to form secure attachments with their caregivers than shelter dogs with staff, suggesting improved social welfare for dogs participating in foster programs. Moreover, a paired attachment test in this study revealed that both fostered and shelter dogs are significantly more likely to proximity seek with a caregiver who is attending to and interacting with them when compared to owned dogs. Fostered and shelter dogs also show a higher rate of disinhibited attachment than owned dogs, meaning that they are unbiased in proximity seeking with their caregiver *versus* an unfamiliar person. This lack of bias may underly the effectiveness of relatively short human interaction interventions in the shelter despite the person being unknown to the dog.

In another study by Thielke & Udell (2019), attachment styles of fostered and shelter dogs were compared to dogs’ cognitive scores and behavior assessments. They found that fostered dogs with secure attachments performed better and were more persistent on cognitive tasks than fostered dogs with insecure attachments. Moreover, securely attached fostered and shelter dogs emitted fewer neurotic behaviors than insecurely attached dogs in the shelter or foster care. In total, these studies reveal that fostered dogs are likely to form secure attachments to their caregivers as dogs are with their owners, which may aid in their behavior, cognition, and perhaps ability to form future secure attachments with adopters.

On the human side of attachment, Powell et al. (2024) found that foster caregivers were significantly more likely to form secure attachments with their fostered pets than avoidant or resistant attachments, which aligns with Thielke & Udell (2019) findings about fostered pets’ attachment styles with their caregivers. Additionally, Reese et al. (2024) found that caregivers reported similar levels of emotional attachment to their fostered dogs as those with owned dogs. While preliminary evidence suggests that fostering offers several health and emotional benefits to caregivers and their families, it is possible that caregivers can become overly attached to their fostered pet. Caregivers interact, care for, and nurture their fostered pet while often putting aside their own attachment when the animal is adopted (Roemer, 2004; Thielke & Udell, 2020). Previously, foster caregiving has been described as a form of high-stakes volunteerism when considering the emotional costs to the caregiver and the risks of attachment (Gunter et al., 2023, 2022; Reese, 2021).

For individuals without foster caregiving experience, placement of a caregiver’s first fostered animal may be emotionally challenging. New foster caregivers with no pre-existing relationship with the shelter are four times more likely to adopt their fostered dog than caregivers who previously fostered or were associated with the animal shelter (Gunter et al., 2022). Nevertheless, secure attachments to fostered pets have been found to reduce caregivers’ stress when fostering animals with behavioral or physical needs and positively affect future foster caregiving (Reese et al., 2022b). Despite foster caregivers reporting a prominent feeling of loss at the end of fostering, this feeling has not been reported to affect subsequent caregiving (DeWitt, 2020). While much more research is needed on the subject, individuals who provide foster care and adopt their fostered pet may be more akin to potential or trial adopters than caregivers who successfully place their fostered pet in a new home (Gunter et al., 2022).

### Developing foster programs

Efforts have been made to better understand caregivers’ motivations to provide foster care to companion animals. In a cross-sectional study by Ackerman et al. (2023) that surveyed 131 foster caregivers from five U.S. shelters, most foster caregivers reported being driven to this form of volunteerism in order to provide an animal with love and positively impact their community. Younger foster caregivers (ages 18 to 29) were motivated by companionship, emotional support, and the opportunity to meet new people while adult caregivers (30+) wanted to foster because of companionship, physical activity, and the desire to create more connections in their community. Female-identifying caregivers were more motivated to foster for emotional support than those that were male-identifying. Moreover, canine foster caregivers as well as caregivers without resident pets were found to be more motivated by animal companionship than caregivers fostering cats or those with resident pets in their home.

Daily (2021) found similar motivations with foster caregivers. Data from their survey of 85 caregivers indicated that most caregivers were motivated by saving and enhancing animal lives, often because of their love of animals. Foster caregivers often expressed that they were driven to foster, because they saw a great need or could not have a pet of their own. In total, these studies increase our understanding of foster caregiver expectations and motivations, providing insight that animal shelters can utilize to aid their caregiver recruitment efforts.

### Evaluating foster care programs

Evidence increasingly demonstrates the benefits of foster caregiving on aspects of shelter animal and human welfare; however, few studies have reported on the actual prevalence of foster care programs in U.S. animal shelters. Between 2015 and 2016, it was found that less than 10% of dogs in U.S. shelters experienced foster care, and the provision of foster care tended to be biased toward prioritizing dogs with behavioral or medical needs (Patronek & Crowe, 2018). In 2020, nearly 10% fewer shelters and rescues had an active foster care program as compared to 2018 (Maddie’s Fund, 2020). Following the pandemic, the extent of foster care programs in U.S. shelters is not well known.

Even fewer studies have examined the protective factors that aid in the successful recruitment and maintenance of foster caregivers. According to previous evidence, a caregiver’s secure attachment to their foster pet reduces the stress of providing foster care, which likely aids in caregiver retention. Reese et al. (2022b) found that receiving support, resources, and guidance from the animal shelter helps to reduce the negative emotional aspects of foster caregiving and increases shelters’ retention of caregivers. In a survey of U.S. dog foster caregivers, Reese et al. (2024) reported that about 70% of caregivers felt that they had an adequate level of training before providing foster care, which supported their future caregiving. Over 50% of responding caregivers reported receiving training about canine behavioral needs, but under 50% indicated that they had received food, supplies, a foster mentor, support for medical issues, enrichment items, or sufficient communication from the animal shelter. Caregivers suggested that obtaining further training in canine behavior (25%), communication from their animal welfare organization (15%), general dog training guidance (8%), support (7%), and supplies (7%) would help increase their ability to continue fostering animals and their positive association with caregiving (Reese et al., 2024).

In their study of brief outing and temporary fostering programs, Gunter et al. (2023) evaluated the programmatic success of these types of programs based on three ranked variables: (1) the total amount of fostering experiences a shelter provided, (2) number of caregivers who participated, and (3) days needed for the shelter to reach the study’s sample size benchmark (40 participating dogs). The authors found that shelters that allowed members of the community, without a prior relationship with the shelter, to provide outings and fostering, had more successful programs than those that did not. It is possible that allowing individuals, unaffiliated with the shelter, to participate in these programs increases the visibility of both shelter dogs and fostering programs within the community, improving programmatic performance.

### Changes to foster programs during the COVID-19 pandemic

During the COVID-19 pandemic, animal shelters experienced greater interest in adopting and fostering shelter animals. Morgan et al. (2020) report that daily dog adoption requests at shelters increased from roughly 0–50 on average to nearly 200 per day during the COVID-19 pandemic. The ASPCA reported a 70% increase in shelter animals placed into foster care during the pandemic in Los Angeles and New York as well as a 66% increase in foster caregiving applications nationally (Oakes, 2020; Witten, 2020). Anecdotal reports attributed these responses to “stay-at-home” mandates during the pandemic, which allowed foster caregivers to remain in their residences and spend time with their fostered pet, whereas more traditional employment before the pandemic may have limited their ability to volunteer as a foster caregiver (Szydlowski & Gragg, 2020).

During this time, researchers sought to understand changes to companion animal foster caregiving as well as if interest in caregiving would persist beyond the pandemic. In their national survey of over 600 animal shelters, Reese et al. (2022a) discovered that foster care programs grew, and the number of foster care placements increased during the pandemic. Morgan et al. (2020) also found that the number of foster caregivers during the pandemic increased so significantly for many shelters in Israel that the number of available foster homes often exceeded the need. Nevertheless, Powell et al. (2021) reviewed changes to intake, adoption, euthanasia, and foster care in 14 U.S. shelters from March 2019 to June 2020 and found that adoption, foster care placement, and euthanasia did not significantly change for these shelters during the COVID-19 pandemic; however, the number of dogs and cats brought into them was significantly lower. Despite shelters hoping to expand their foster care programs during the pandemic, they found that the proportion of fostered animals did not significantly increase.

A study by Gunter et al. (2022) assessed changes to dog foster caregiving programs at 19 U.S. animal shelters between March and June 2020. They measured the number of dogs residing in the shelter and foster care daily in order to calculate shelters’ utilization of fostering. The authors found that the proportion of animals in foster care relative to those living in the shelter significantly increased between March and April 2020; however, foster utilization returned to pre-pandemic proportions by June 2020. For municipal shelters, this decrease in foster caregiving occurred more quickly than for private nonprofit shelters as well as private nonprofits with municipal contracts. While foster programs likely grew during the COVID-19 pandemic due to stay-at-home orders, increases in fostering were likely short-lived after caregivers returned to their jobs and resumed pre-pandemic activities outside of the home.

It is worth noting that Gunter et al. (2022) found two adoption practices during the pandemic that were weakly but significantly associated with fewer days in foster care for shelter dogs. They found that when sheltering organizations allowed potential adopters to meet fostered dogs at the homes of their caregivers, these dogs had shorter lengths of stay than dogs at shelters that did not allow such meetings. Additionally, Gunter et al. (2022) uncovered that when shelters allowed adopters to pick up their dogs directly from foster homes, these dogs had shorter lengths of stay than dogs at shelters that did not allow for this type of caregiver-facilitated acquisition. The authors suggest that these practices may be a form of open adoption, from the organization’s perspective, by decentralizing placement and empowering foster caregivers to perform adoptions based on the information and experiences they have had with their fostered dog.

While euthanasia likely decreased and adoption and foster placements increased during the COVID-19 pandemic, these positive changes in animal sheltering were likely temporary. Carroll, Reeve & Torjussen (2024) found that post-pandemic, shelters in the UK have observed increases in relinquishment as well as cases of animal abuse and severe animal neglect. While pandemic adopters sought companionship during this unprecedented time, perhaps similarly to foster caregivers, many owners indicated that they could no longer care for adopted pets and provide for their behavioral needs after workplaces, schools, and personal schedules returned to normal. This aligns with quantitative reports about U.S. animal sheltering for 2023, wherein the total number of dogs adopted decreased by 5% in comparison to 2019, and 4% more animals entered shelters than in 2021 (Shelter Animals Count, 2023).

### Challenges and obstacles in foster caregiving

As with any service, foster caregiving is not without its challenges. Experiencing difficulties during foster care can create emotional drawbacks for caregivers. As such, the duration and frequency of foster caregiving can vary; some caregivers provide care only once, while others do so repeatedly (Chur-Hansen et al., 2014; Gunter et al., 2019). A study of Brazilian foster homes indicated that 28.7% of caregivers were first-time caregivers, while 26% of caregivers had provided foster care to more than 10 animals (Taraciuk et al., 2020).

High levels of emotional stress can contribute to thoughts of quitting and elements of compassion fatigue, including burnout and secondary traumatic stress (Reese et al., 2022b; Chur-Hansen et al., 2014; Daily, 2021; DeWitt, 2020; Roemer, 2004). Foster caregivers of animals with medical or behavioral issues are especially likely to experience emotional challenges. In a study by Reese et al. (2022b), the authors found that nearly a quarter of foster caregivers indicated that fostering animals with behavioral issues is emotionally difficult and draining, while 7% of caregivers reported that fostering animals with medical needs was emotionally challenging. In their examination of fostering during the pandemic, Gunter et al. (2022) found that adult dogs with known behavioral issues were over three times more likely to be returned from foster care for behavioral reasons than dogs without behavioral issues, highlighting the challenges inherent in providing this type of foster care.

In an effort to better understand factors associated with caregiver retention, Reese et al. (2024) queried U.S. dog foster caregivers and received responses from 611 caregivers regarding their reasons for no longer fostering. Many caregivers (74%) discontinued, because their schedule was no longer compatible with fostering. About 50% of respondents mentioned burnout, lack of support from their animal shelter, and issues between fostered dogs and family members. Less than 30% ceased caregiving due to adopting their fostered dog or having a negative experience with a fostered dog. Similarly, when Daily (2021) surveyed dog foster caregivers to assess their experiences, including the most negative aspects of fostering, 17% of caregivers indicated they experienced struggles with their fostered dogs’ behavioral needs that made these dogs challenging to live with or be adopted. Another 9% of caregivers specifically reported difficulties with fostered dogs disrupting their home and routine. A portion of respondents (7%) also described challenges managing the fostered dog with resident dogs in the home. Seeing fostered dogs in pain (14%) and dealing with medical, health, and age-related issues (10%) were indicated as well, suggesting that caregivers may struggle with secondary trauma.

In the Daily (2021) study, caregivers reported other difficulties, including a spectrum of emotions associated with placing their fostered dog with its adopter after forming an attachment (13%) as well as an inability to rehabilitate or place their dog (7%). Powell et al. (2024) found that over three-quarters (78%) of U.S. foster caregivers indicated feeling some level of grief or missing their fostered pet after it was adopted, and 30% reported tearful periods since their fostered pet’s departure. Foster caregivers in Brazil reported similar challenges to fostering animals as indicated by Daily (2021), including finding adopters for their fostered pets (81.3%), the financial burden of fostering (54%), difficulties with the fostered animal’s behavior (30.7%), family hesitations or resistance to fostering (26.7%), and having time to care for fostered animals (24%; Taraciuk et al., 2020).

### Discrepancies in equity and diversity of foster care programs

Animal shelters with varying levels of resources use different methods to recruit foster caregivers, creating programmatic discrepancies. Lower-resourced shelters tend to rely on pre-existing relationships with caregivers to meet their organization’s fostering needs. Conversely, shelters with greater resources more often utilize new caregivers from their surrounding community, likely due to these organizations’ ability to spend time recruiting and training new foster caregivers. This disparity can lead to greater advantages for well-resourced shelters in foster program development, maintenance, and expansion (Gunter et al., 2022).

In addition to differences in shelter resources, discrepancies exist in the diversity of foster caregivers and the programs studied thus far. Samples from previous studies about companion animal caregiving have described foster caregiver and shelter volunteer populations as predominantly white, female-identifying, and adult (under 65) with the most studied foster care programs located in North America (Reese et al., 2022b; Graham et al., 2024; Roemer, 2004; Daily, 2021; Taraciuk et al., 2020; Neumann, 2010). In the U.S., barriers to owning pets, including housing and public transportation that are not pet-inclusive, disproportionately discourage lower-income households from participating in shelter foster care programs (McLennan et al., 2022; Toohey & Krahn, 2018). Additionally, low recruitment and inclusion of marginalized populations over time have likely contributed to a homogeneous population of foster caregivers within animal sheltering that has little diversity (Ly, Gordon & Protopopova, 2021; Gandenberger et al., 2021).

To date, no study has explored foster care programs in Indigenous communities and only one study has reviewed an animal fostering program in a largely Hispanic/Latinx community, where the authors found that individuals were often unaware of existing foster programs at their local animal shelters (McDonald et al., 2022). The current literature on companion animal fostering highlights inherent issues of inclusivity within animal sheltering and the need for animal welfare organizations to fully engage with their community to fulfill their lifesaving missions. Reporting community representation and how that representation is reflected in foster caregiving programs is a much-needed addition to future research studies.

### Best practices & recommendations for foster care programs

Current scientific literature continues to provide promising evidence for the welfare benefits of foster care for both animals and humans. Brief outings from the animal shelter offer opportunities to improve the distal welfare of shelter dogs by significantly increasing their likelihood of adoption: five times greater than dogs that do not participate in such programs. Temporary foster care, even for a single night, improves both dogs’ proximate and distal welfare, such that their likelihood of adoption increases by over 14 times (Gunter et al., 2023) as well as significantly reducing canine stress and increasing rest (Gunter et al., 2019). As such, dogs that are struggling behaviorally in the animal shelter would be best supported by a fostering stay, not a brief outing into the community.

Caregivers gain emotional and social benefits from providing foster care to shelter animals, including experiences of companionship, affection, and emotional support, which increases their positive affect and decreases feelings of loneliness and depression (Powell et al., 2024; Sanderson et al., 2023). The physical welfare of caregivers is also improved through fostering as indicated by increased heart rate variability and activity (Ortmeyer & Robey, 2019; Potter et al., 2019).

Nevertheless, fostering requires that caregivers care for their fostered pet while also yielding their attachment when the animal is adopted (Roemer, 2004; Thielke & Udell, 2020). While caregivers do adopt their fostered pets, it is unlikely and might be related to the duration of caregiving with longer durations of fostering associated with higher rates of adoption (but still below 20%). It is especially important for caregivers of dogs with behavioral issues to receive organizational support as the needs of these dogs are more involved than typical foster care and may negatively impact caregivers’ mental health and their fostering experience (Gunter et al., 2023; Reese et al., 2022b).

Utilizing a stepwise approach to fostering can support the retention of caregivers due to the high-stakes nature of this type of volunteerism. Allowing new caregivers to begin with shorter durations of foster care will likely increase their willingness to provide foster care in the future as the rewards of caregiving easily exceed the physical and emotional costs. As these rewards are experienced through multiple fostering stays, riskier fostering opportunities of longer durations or with animals that have more significant behavioral or medical needs could be attempted. Throughout, shelters need to support foster caregivers by providing them adequate resources, instruction, and guidance to reduce the negative aspects of fostering and increase caregiver retention (Reese et al., 2022b).

Moreover, first-time foster caregivers with no relationship to the shelter, particularly those with no dogs in their home, and those who are fostering-to-adopt (trial adopters) are quite possibly more alike than dissimilar in their adoption likelihood. As such, it may behoove animal shelters to have comparable onboarding processes for these two groups, such that new caregivers can initially foster dogs available for adoption and receive similar preliminary support as those in foster-to-adopt programs. If first-time caregivers do not adopt during their initial caregiving experience (in which they are most likely to do so), organizations can then provide more in-depth caregiver training in preparation for future fostered pets. This strategic approach may be of particular benefit to lesser resourced shelters and better align post-placement support services for caregivers and adopters.

Allowing caregivers to meet with prospective adopters and adopters to pick up their new pets, both at the caregivers’ homes, are two practices that have been associated with shorter lengths of stay in foster care. It is possible that these practices can help speed the adoptive placement of fostered animals, allow organizations to meet the fostering needs of more shelter animals, and provide greater empowerment to foster caregivers. Additionally, animal shelters should consider a community-inclusive option in their foster care programs. With this option, individuals with no relationship to the shelter can easily participate in same-day caregiving, including brief outings or temporary foster care for shelter dogs that are behaviorally and medically sound, as this has been shown to increase the performance of short-term fostering programs (Gunter et al., 2023).

### Limitations

While this review presents promising evidence regarding the impacts of foster care programs on the lives of shelter animals and their caregivers, it is worth highlighting the limitations of this review. In accordance with the geographic focus of the current literature about companion animal foster caregiving, this review evaluates interventions largely based in the United States and Canada, with little representation from programs in South America, Asia, and Australia. While foster care likely exists in these regions, their exclusion from the scientific literature leaves little to report in this review. Additionally, the reported impacts of animal fostering for caregivers have been investigated through experiences of primarily white, female-identifying individuals in the U.S., Canada, and Europe. Similarly, we recognize our bias towards English-language publications in this review. As such, the samples utilized in these studies may reduce generalizability to a more diverse group of foster caregivers. It is also worth noting that this review, like the current literature on foster care programs, is largely focused on the impacts of foster care systems on shelter dogs, with a clear gap in examinations of fostering programs for cats and small animals.

## Conclusions

This review of current literature on foster care programs has consistently concluded that fostering improves the distal (*i.e*., length of stay and adoption outcomes) and proximate (*i.e*., physiological and behavioral) welfare of shelter animals. Despite these clear benefits, as of 2020, only about 78% of animal welfare organizations in the United States have a foster care program, and the extent that these programs are experienced by animals is not well understood (Maddie’s Fund, 2020). Based on research by Gunter et al. (2023), it is likely that resources play a role in the success of fostering programs. In their study, Gunter et al. found that the available resources of an organization are influential in the success of short-term dog fostering programs, such that shelters with greater resources are higher performing than those with fewer resources. As such, if organizations are considering implementing or expanding their existing foster programs, it is incumbent that they provide the requisite human and financial resources to carry out these programs to positively impact the lives of shelter animals.

Evidence suggests that foster care programs can offer numerous benefits to companion animal welfare and the mental, physical, and emotional well-being of foster caregivers. However, providing foster care is not without its difficulties for caregivers, which can negatively impact their well-being; as such, these challenges should be considered alongside its benefits. Meeting the needs of the fostered animal, managing interactions with household members (including resident pets), balancing scheduling and home circumstances, and overcoming attachment once a fostered animal is adopted are all challenges that can contribute to high emotional stress for caregivers, reducing their retention. Emotional stress is particularly salient for foster caregivers of animals with medical or behavioral concerns. While foster caregivers may experience an increase in activity, sense of purpose, companionship, and improvements to their social and emotional health while providing foster care, they may also experience stress, grief, or burnout during or after caregiving.

The scientific literature about foster care programs for shelter dogs has grown and includes promising, consistent evidence about the importance of these programs for canine welfare. In contrast, literature on cat foster programs has been less prevalent, with mixed results as to their impacts on feline proximate and distal welfare. Future studies that delve into specific populations of cats (*i.e*., older cats, kittens, cats with medical/behavioral needs) and longer durations of foster care, may help crystallize our understanding of how foster care can further aid cats in need of homes. Additionally, foster care research regarding other companion animals, such as “pocket pets” (*e.g*., guinea pigs, rabbits, rats), has yet to be explored and would also be a beneficial contribution to knowledge in this domain.

Current evidence indicates that the most successful companion animal foster care programs are ones in which foster caregivers feel physically and emotionally prepared to provide care. Whether shelters are implementing new programs or intending to grow existing ones, organizations should consider providing caregivers with the necessary supplies and training as well as instrumental and informational support. These may include consistent communication with shelter staff who are knowledgeable in animal health and behavior, support groups (*e.g*., peer-to-peer or shelter-led), or counseling services, both for themselves or their fostered pet, to utilize as they confront challenges during their foster care experiences. Developing a stepwise approach to caregiving engagement, in which new caregivers begin with shorter periods of fostering and increase their duration of caregiving over time, is a promising way to support foster caregivers and potentially increase retention.

Finally, foster care programs should seek to engage with more individuals from historically marginalized communities, Indigenous populations, and people within geographic areas of the community who have been typically less involved with the animal shelter. Doing so may provide animal welfare organizations with further possibilities to extend their placement networks as well as create more inclusive, equitable practices to advocate for people and their pets. Taken together, supporting foster caregivers and increasing the diversity and extent of community engagement remain opportunities where animal shelters can extend their companion animal fostering programs. Moving forward, lesser-researched components of these programs may be some of the more promising ways we can further expand our understanding of foster caregiving in animal shelters.

## Additional Information and Declarations

### Data Availability

The following information was supplied regarding data availability: This is a literature review.
